# Investigation of *Cryptococcus neoformans* magnesium transporters reveals important role of vacuolar magnesium transporter in regulating fungal virulence factors

**DOI:** 10.1002/mbo3.564

**Published:** 2017-12-15

**Authors:** Chen‐Hao Suo, Lan‐Jing Ma, Hai‐Long Li, Jian‐Fang Sun, Chao Li, Ming‐Hui Lin, Tian‐Shu Sun, Wei Du, Yan‐Jian Li, Xin‐Di Gao, Yang Meng, Si‐Xiang Sai, Chen Ding

**Affiliations:** ^1^ College of Life and Health Sciences Northeastern University Liaoning Shenyang China; ^2^ School of Medicine Binzhou Medical University Yantai Shandong China; ^3^ Present address: Department of Scientific Research Central Laboratory Peking Union Medical College Hospital Chinese Academy of Medical Sciences Beijing China

**Keywords:** Capsule, *Cryptococcus neoformans*, melanin, Mg homeostasis

## Abstract

*Cryptococcus neoformans* is an important opportunistic fungal pathogen in humans. Recent studies have demonstrated that metals are critical factors for the regulation of fungal virulence in hosts. In this study, we systemically investigated the function of *C. neoformans* magnesium transporters in controlling the intracellular Mg balance and virulence‐associated factors. We identified three Mg transporters in *C. neoformans*: Mgt1, Mgt2*,* and Mgt3. While we could not detect a Mg^2+^‐related growth phenotype in *mgt1* and *mgt3* knockout strains, a *GAL7p‐*Mgt2 strain showed significant Mg‐dependent growth defects in the presence of glucose. Further analysis demonstrated that *MGT2* is a homolog of *MNR2* in *Saccharomyces cerevisiae,* which is localized to the vacuolar membrane and participates in intracellular Mg transport. Interestingly, a transcriptome analysis showed that Mgt2 influenced the expression of 19 genes, which were independent of Mg^2+^. We showed that melanin synthesis in *C. neoformans* required Mg^2+^ and Mgt2, and that capsule production was negatively regulated by Mg^2+^ and Mgt2. Repressing the expression of *MGT2‐*induced capsule, which resulted in an increased fungal burden in the lungs. Cumulatively, this study sets the stage for further evaluation of the important role of Mg homeostasis in the regulation of melanin and capsule in *C. neoformans*.

## 1 INTRODUCTION

*Cryptococcus neoformans* is a major fungal pathogen in humans. It primarily afflicts immunodeficient and immunocompetent individuals, causing about 600,000 deaths per year globally (Idnurm et al., 2005; Kronstad et al., 2011; May, Stone, Wiesner, Bicanic, & Nielsen, 2016). *Cryptococcus* infections begin in the lung tissue. By unknown mechanisms, fungal cells disseminate into the central nervous system, resulting in lethal meningitis (Idnurm et al., 2005; Kronstad et al., 2011; May et al., 2016). Recent epidemiological studies have revealed that infections of *Cryptococcus* species are a significant health issue in the Pacific Northwest in Northern America, in addition to Europe, Africa, and China (Byrnes et al., 2010, 2011; Liu et al., 2017; May et al., 2016).

Two major fungal virulence factors have been extensively studied. These are the generation of a cell‐surface polysaccharide capsule structure and melanization using L‐DOPA as a substrate (Idnurm et al., 2005; Kronstad et al., 2011). The capsular structure is critical for shedding the fungal cells from the host immune cells and manipulating the responses of immune cells to *C. neoformans* invasion (May et al., 2016; Zaragoza et al., 2009). Melanin formation in *C. neoformans* protects fungal cells from many environmental stresses, such as UV radiation and free‐radical toxicity (Wang & Casadevall, 1994; Williamson, 1997). Numerous studies have demonstrated that metals, such as copper (Cu) and iron (Fe), regulate the production of the capsule structure and melanin pigment (Bairwa, Hee Jung, & Kronstad, 2017; Chun & Madhani, 2010; Ding, Festa, Sun, & Wang, 2014; Do et al., 2016; Jung, Sham, White, & Kronstad, 2006; Lian et al., 2005). It is known that the key melanin biogenesis protein (Lac1) utilizes Cu^+^ as a cofactor (Williamson, 1997). Disrupting key regulators of Cu homeostasis of *C. neoformans* influences melanin formation. For example, disrupting the Cu^+^ transporter genes *CTR1* and *CTR4*, the Cu^+^ chaperone *ATX1,* or the P‐type ATPase *CCC2* impairs the development of dark brown pigments in *C. neoformans* (Chun & Madhani, 2010; Ding et al., 2011; Walton, Idnurm, & Heitman, 2005; Waterman et al., 2007, 2012). Fe, the most well studied metal in *C. neoformans*, plays extremely important roles in modulating melanin and the capsule (Jung et al., 2006; Lian et al., 2005; Tangen, Jung, Sham, Lian, & Kronstad, 2007). A master Fe homeostasis regulator in *C. neoformans*, transcription factor Cir1 was demonstrated to inhibit melanin formation by repressing expression of *LAC1* and *LAC2*. It also induces capsule biogenesis by activating capsule production‐related genes (Jung et al., 2006). Moreover, *C. neoformans* cells utilize melanin as a Fe reduction system (Nyhus, Wilborn, & Jacobson, 1997). Given the important functions of these metals in regulating fungal virulence factors, human fungal pathogens have evolved sophisticated acquisition and defense machineries to cope with metal deficiency and overload conditions at the pathogen‐host axis (Bairwa et al., 2017; Ding et al., 2014).

Of all‐important metals in biology, magnesium (Mg) is one of the most abundant metals in the cell and it plays critical roles as an enzymatic cofactor and structural component of biological molecules. Many mammalian disorders are associated with Mg^2+^ imbalance in the cell, such as depression, anxiety, agitation, and insulin resistance (Abdulatif, Ahmed, Mukhtar, & Badawy, 2013; Boyle, Lawton, & Dye, 2016, 2017; Serefko, Szopa, & Poleszak, 2016; Serefko et al., 2013). In fungal cells, supplementation of Mg^2+^ in the growth medium protects *Saccharomyces cerevisiae* cells from dehydration (Trofimova, Walker, & Rapoport, 2010). Mg^2+^ is also involved in mitotic spindle formation in *Schizosaccharomyces pombe* (Uz & Sarikaya, 2016). In *S. cerevisiae*, the Mg^2+^ transporters Alr1 and Alr2 were identified as plasma membrane‐bound Mg importers. The knockout strains for *ALR1* and *ALR2* display blocked Mg^2+^ uptake, which reduces intracellular Mg^2+^ concentrations and subsequently inhibits cell growth in normal growth condition (da Costa, Cornish, & Keasling, 2007). *S. cerevisiae* Mnr2 is a vacuolar Mg^2+^ transporter that pumps Mg^2+^ from the vascular lumen into the cytosolic space (Pisat, Pandey, & Macdiarmid, 2009). Mg^2+^ is required for mitochondrial function in fungi. Three mitochondrial Mg^2+^ transporters have been identified in *S. cerevisiae*. Lpe10 and Mrs2 are Mg^2+^ importers and Mme1 acts as a mitochondrial Mg^2+^ exporter. Inactivation of any mitochondrial Mg^2+^ transporters reduces Mg^2+^ accumulation in the mitochondria and causes defects in respiration (Cui et al., 2015; da Costa et al., 2007).

Although Mg^2+^ homeostasis plays vital functions in fungal biological processes, the role of Mg^2+^ in regulating virulence factors in human fungal pathogens remains largely unknown. In this study, we examined the function of three *C. neoformans* Mg^2+^ transporters with respect to the regulation of fungal virulence‐associated factors. We demonstrated that a vacuolar Mg^2+^ transporter (Mgt2) in *C. neoformans* positively regulated Mg^2+^ delivery and melanin formation, but negatively controlled capsule production. Using transcriptome analysis, we showed that knockdown of *MGT2* differentially regulated the expression of 118 genes. Of these, 19 genes were influenced by *MGT2* knockdown independently of exogenous Mg. In animal infection models, the *mgt2* mutant displayed an elevated fungal burden in lung tissues as a result of an enlarged capsular structure.

## 2 MATERIAL AND METHODS

### 2.1 Fungal strains and growth conditions

The wild‐type strain H99 was used to generate Mg transporter mutants. Mutants were generated by homologous recombination and transformed using ballistic transformation as described previously (Toffaletti, Rude, Johnston, Durack, & Perfect, 1993). Fungal cells were grown in YPD medium (1% yeast extract, 2% peptone, 2% glucose) at 30°C. To activate the *C. neoformans GAL7* promoter, YPGal was used (1% yeast extract, 2% peptone, 2% galactose). After biolistic transformation, fungal cells were plated onto YPDSorb plates (1% yeast extract, 2% peptone, 2% galactose, 2% agar, 1 mol/L sorbitol). The transformant selection was performed using YPD agar supplemented with 200 μg/ml of G418. A final concentration of 50 mmol/L MgSO_4_ was used for *GAL7p‐MGT2* cell growth.

### 2.2 Plasmid construction and mutant construction

The generation of *C. neoformans* strains in which Mg transporter genes were disrupted was performed as follows. All primers used for the generation of mutants are listed in Table S1. For the targeted disruption of the *MGT1* or *MGT3* open reading frame, the upstream sequence of *MGT1* or *MGT3* was amplified using primer pair CDp280/CDp281 or CDp288/CDp289, respectively. The PCR products were cloned between the *Apa*I and *Xma*I restriction sites in a *NEO* ^*R*^ plasmid. The downstream sequence of *MGT1* or *MGT3* was amplified using primer pair CDp282/CDp283 or CDp290/CDp291, respectively. The resulting PCR products were cloned between the *BamH*I and *Xba*I restriction sites of the *NEO* ^*R*^ plasmid. The resulting plasmids were sequenced, and the inserts were amplified using primer pair CDp23/CDp24 to generate the knockout integration cassette. The PCR products were purified, concentrated, and delivered using a PDS‐1000/He System (Bio‐rad). Transformants were picked and genomic DNA was isolated for PCR confirmation using primer pair CDp428/CDp131 for *MGT1* and CDp962/CDp131 for *MGT3*.

The *C. neoformans mgt2* mutant was constructed by replacing the *MGT2* endogenous promoter with the *GAL7* promoter. The upstream sequence from *MGT2* was amplified using primers CDp284 and CDp285, and then this was cloned between the *Apa*I and *Xma*I restriction sites in a *NEO* ^*R*^ plasmid. The *C. neoformans GAL7* promoter or *MGT2* ORF (first ~1 kb of genomic DNA sequence) were amplified using primer pair CDp611/CDp612 or CDp613/CDp614, respectively. The resulting PCR fragments were purified and mixed, and overlapping PCRs were performed to join the *GAL7* and *MGT2* fragments using primers CDp611 and CDp614. The overlapping PCR product was purified and cloned between the *Spe*I and *Sac*II restriction sites in the *NEO* ^*R*^ plasmid. The integration cassette was amplified using primers CDp23 and CDp24. The PCR fragment was transformed into *C. neoformans* by biolistic transformation. After 4 hr of recovery incubation, cells were transferred onto YPGal agar supplemented with G418. Transformants were confirmed by PCR using primers CDp632 and CDp131 and by Southern blotting.

For the construction of an overexpression strain of Mgt2, the *C. neoformans TEF1* promoter was used to constitutively drive gene expression at the *CMT2* locus. The upstream or downstream sequence of *CMT2* was amplified from genomic DNA using primer pair CDp732/CDp733 or CDp738/CDp739, respectively. The upstream fragment was then cloned between the *Hind*III and *EcoR*I restriction sites in a *NAT* ^*R*^ plasmid, and the downstream fragment was cloned between the *Xba*I and *Sac*II restriction sites in the same plasmid. The *C. neoformans TEF1* promoter was amplified using primers CDp2204 and CDp2205, and the *MGT2* ORF was amplified using primers CDp2206 and CDp2207. The fragments of the *TEF1* promoter and the *MGT2* ORF were joined by overlapping PCR using primers CDp2204 and CDp287. The resulting fragment was cloned at the *Xba*I site in the plasmid. The integration cassette was transformed using biolistic transformation. Successful integration at the *CMT2* locus was confirmed using primers CDp861 and CDp131.

For the expression of *S. cerevisiae ALR1* or *MNR2* in the *GAL7‐MGT2* strain, a *C. neoformans GPD1* expression plasmid (pXL‐HYG) was used as described previously. The genomic DNA sequence from *S. cerevisiae ALR1* or *MNR2* was amplified using primer pair CDp1699/CDp1700 or CDp1954/CDp1955, respectively. The resulting PCR products were cloned into the *Pac*I site in the pXL‐HYG plasmid. The plasmids with the correct gene orientation were transformed in the *C. neoformans GAL7‐*MGT2 strain.

### 2.3 Genomic DNA isolation and Southern blotting

Genomic DNA isolation and Southern blotting were performed as previously described (Ding & Butler, 2007). Briefly, 5 ml of cell culture (YPD medium or YPGal medium) was washed twice with water and then resuspended in lysis buffer (100 mM Tris‐HCl pH 8.0, 50 mmol/L EDTA, 1% SDS, 100 mM NaCl, 2% Triton X‐100). Cells were disrupted using a bead beater (Biospec). The supernatant was mixed with 7 mol/L ammonia acetate (pH 7.0). Genomic DNA was extracted using chloroform and precipitated with isopropanol. DNA pellets were then washed, dried, and resuspended in water. Next, 1 μg of DNA was used for PCR. Genomic DNA (20 μg) was treated with RNase A and digested with *Stu*I and *Sac*II overnight for Southern hybridization analysis. Southern blotting was performed using a DIG High Prime DNA Labeling and Detection Starter Kit II (Roche), and the experimental procedure was carried out according to the manufacturer's manual. The DIG‐labeled DNA probe was synthesized by PCR using primers CDp1045 and CDp612 (Table S1).

### 2.4 RNA isolation, real‐time PCR, and transcriptome analysis

RNA samples used for quantitative real‐time PCR and transcriptome library construction were isolated using TRIzol reagent (Thermo Fisher Scientific) followed by Turbo DNase I treatment (Thermo Fisher Scientific) to eliminate DNA contamination. Next, 1 μg of total RNA was reverse‐transcribed into cDNA using the Go Script Reverse Transcription System (Promega). Real‐time PCR was performed using a CFX Connect Thermocycler (Bio‐Rad). The data were analyzed using 2^−ΔΔCt^ method. *ACT1* was used as a loading control. Primers are listed in Table S1. For RNA‐seq analysis, 3 μg of total RNA were processed using the TruSeq RNA Sample Preparation Kit (Illumina, San Diego, CA, USA). Next, mRNA was purified from total RNA using the polyT oligo‐attached magnetic beads. An Illumina proprietary fragmentation buffer was used for mRNA fragmentation. First‐strand cDNA was synthesized with random hexamer primers using SuperScript II followed by second‐strand cDNA synthesis using RNase H and DNA polymerase I. Overhang sequences were then blunted and adenylated at the 3′ end of the cDNA sequence. Subsequently, cDNA sequences 200 bp in length were selected using the AMPure XP system (Beckman Coulter) and enriched using Illumina PCR Primer Cocktail in a 15‐cycle PCR reaction. The PCR products were purified and their integrity was confirmed using the Agilent high sensitivity DNA assay on a Bioanalyzer 2100 (Agilent). The sequencing library was then sequenced on a Hiseq platform (Illumina) by Shanghai Personal Biotechnology Cp. Ltd. Sequenced reads were mapped using TopHat (http://ccb.jhu.edu/software/tophat/), and the transcript assembly was performed using Cufflinks (http://cufflinks.cbcb.umd.edu) (Trapnell et al., 2012). Genes with differential expression were analyzed using DESeq, as previously described (Chen et al., 2014). Genes of the mutant strains with fold changes greater or less than 1.5 in comparison to that of the wild‐type strain were considered to be upregulated or downregulated, respectively.

### 2.5 Microscopy

The capsule was observed using a Nikon Eclipse Ni‐E microscope with the 100× objective (Nikon). Lung tissue mucicarmine and hematoxylin/eosin staining were visualized using a Leica DM4000B LED microscope (Leica) with the 20× objective. Protein localization of GFP‐Mgt2 was performed by inducing cells in medium supplemented with galactose and 200 μM EDTA for 6 hr. The GFP signal was observed using a Leica TCS‐SP8 confocal microscope under the 65× oil objective (Leica).

### 2.6 Animal virulence assay, fungal burden, and histopathology

All animal experiments were reviewed and ethically approved by the Research Ethics Committees of the College of Life and Health Sciences of Northeastern University. The animal infection experiments were performed as previously described (Sun et al., 2014). Briefly, 6–8‐week‐old female C57BL/6 mice were used for survival rate and fungal burden analysis, and they were purchased from Changsheng Biotech (China). For the survival rate assay, mice were anesthetized and intranasally administered 10^5^ fungal cells in 50 μl of PBS. Animals were monitored twice a day for morbidity. For the fungal burden analysis, infected mice at 14 days post infection were killed by exposure to CO_2_. Lung and brain tissues were collected, weighed, and homogenized in 5 ml of PBS. The suspensions were diluted with PBS, and serial dilutions were plated onto YPGal agar at 30°C for 3 days. For the histopathological assay, lung tissues were collected at 14 days post infection and fixed with paraformaldehyde. Fixed lung tissues were frozen and processed using a Crypstat Microtome Leica CM1850 (Leica). Tissue sections (10 μm in thickness) were stained with mucicarmine or hematoxylin/eosin (Sigma).

### 2.7 Melanin formation and capsule induction

Melanin was quantified using L‐DOPA agar medium. Overnight cell cultures were diluted to an *A*_*600*_ of 1.0 and spotted onto L‐DOPA plates supplemented with various concentrations of MgSO_4_ (0 g/L, 0.25 g/L, 0.5 g/L, or 1 g/L). The plates were incubated at 37°C until black pigment was observed. Photographs were then taken. The capsule induction was performed using two induction conditions. Cells were either incubated in DMEM supplemented with 10% FCS at 37°C and 5% CO_2_ for 3 days, or in ACSF medium supplemented with 0.1% glucose at 37°C for 3 days (Rathore, Raman, & Ramakrishnan, 2016). The capsule was stained with India ink.

### 2.8 Atomic absorption spectroscopy

To quantify intracellular Mg concentrations, the wild‐type and *Gal7p‐MGT2* cells were inoculated in YPGal medium overnight. The cultures were diluted to an *A*_*600*_ of 0.2 in 10 ml of YPD medium and incubated on an orbital shaker at 30°C for 6 hr. Cells were then washed twice with 10 ml of 1 mmol/L EDTA and twice with deionized water to remove Mg ions from the medium and cell surface. The washed cells were collected in preweighed Eppendorf tubes, and the tubes were incubated at 80°C overnight to eliminate residual water. The dried cells were weighed. Next, 400 ml of 70% nitric acid was added to digest the cells at 100°C for 30 min. The resulting suspensions were diluted with 1% nitric acid. Mg concentrations were quantified using a JENA Atomic Absorption Spectroscopy ZEE nit 700P (JENA). The operation procedure was performed according to the manufacturer's manual. A standard curve was first constructed using an Mg atomic spectroscopy standard solution (Sigma). The Mg concentrations were then calculated using the standard curve.

## 3 RESULTS

### 3.1 Identification *C. neoformans* Mg transporters

**Figure 1:**
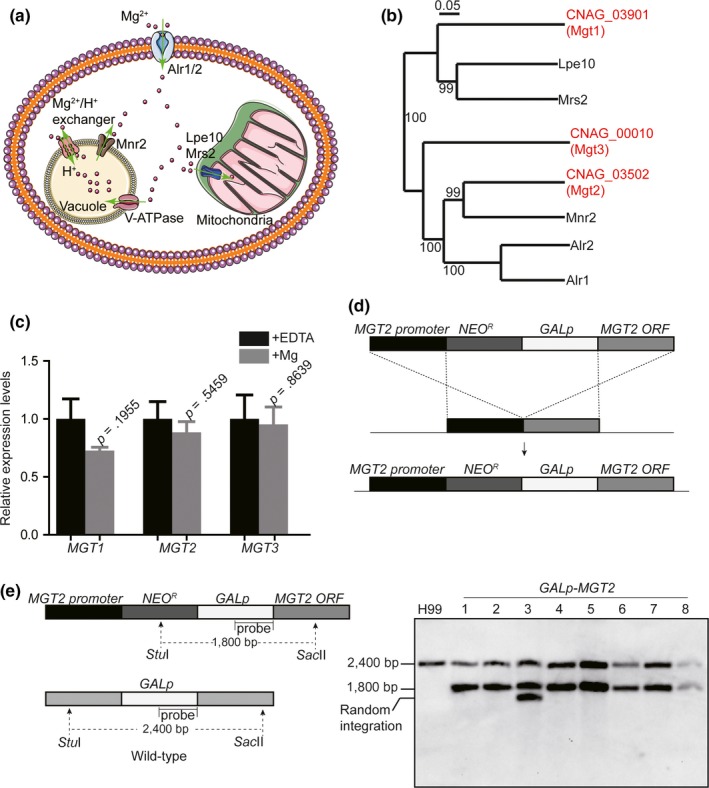
Identification of Mg transporters and generation of knockout mutants in *C. neoformans*. (a) Mg homeostasis in *S. cerevisiae*. The genome of baker's yeast encodes two Mg importers, Alr1 and Alr2 (Da Costa et al., 2007; Graschopf et al., 2001). Mnr2 is a vacuolar Mg transporter that pumps Mg into the cytosol (Pisat et al., 2009). A Mg^2+^/H^+^ exchanger also participates in Mg transport into the cytosol. Mg ions are delivered into the mitochondria via the Lpe10 and Mrs2 transporters (Gregan et al., 2001; Sponder et al., 2010). (b) Phylogenetic analysis of Mg transporters in *S. cerevisiae* and *C. neoformans*. A phylogenetic tree was generated as previously described (Ding et al., 2011). Numbers indicate the bootstrap resampling. Mg transporters in *S. cerevisiae* are labeled in black and those from *C. neoformans* are labeled in red. (c) Real‐time PCR quantifications of *MGT1*,* MGT2,* and *MGT3*. Overnight *C. neoformans* cell cultures were diluted to an *A*_*600*_ of 0.2 in YPD medium, and the cultures were incubated on an orbital shaker at 30°C until an *A*_*600*_ of 1.0. A final concentration of 50 mmol/L of MgSO_4_ or 200 μM EDTA were added to the cultures, and the incubations continued for 6 hr before RNA samples were isolated. Next, real‐time PCR was performed. Expression levels were normalized to *ACT1*. Four biological samples were used for the analysis. Standard deviations were determined, and the Student's *t‐*test was performed. (d). Scheme showing the construction of the *GAL7p‐MGT2* strain. The construction of the integration cassette harboring a *GAL7* promoter is described in the Material and Methods. Briefly, the promoter and ORF fragments from *MGT2* were amplified using PCR and cloned into a *NEO* ^*R*^ *‐GAL7p* plasmid. The resulting plasmid was introduced into the wild‐type strain using biolistic transformation. (e) Southern blot analysis of *GAL7p‐MGT2* strains. The Southern blot analysis was performed as described in the Material and Methods. A probe for the *GAL7p* promoter was used. The restriction enzymes *Stu*I and *Sac*II were used for genomic DNA digestion. To verify the correct integration of the *GAL7p‐MGT2* cassette, two *GAL7p* DNA species should be detected: one from the integrated cassette (resulting in a 1800‐bp band) and the other from the wild‐type *GAL7p* (resulting in a 2400‐bp band). For the wild‐type strain, only one band was detected at 2400 bp. Eight isolates (including two biological integrations) were used for the analysis. All isolates demonstrated correct integrations except isolate 3, which contained a random integration of the cassette in the genome

Previous studies in baker's yeast identified five Mg^2+^ transporters, including two plasma membrane‐bound Mg transporters (Alr1 and Alr2), a vacuolar Mg^2+^ transporter (Mnr2), and two mitochondrial Mg^2+^ transporters (Lpe10 and Mrs2) (Figure 1a) (Bui, Gregan, Jarosch, Ragnini, & Schweyen, 1999; Cui et al., 2015; da Costa et al., 2007; Pisat et al., 2009). To identify Mg^2+^ transporters in *C. neoformans*, a protein phylogenetic analysis was employed. Three *S. cerevisiae* Mg^2+^ transporter homologs were found in a proteome database for *C. neoformans,* including CNAG_03901, CNAG_03502, and CNAG_00010. These were re‐named Mgt1, Mgt2, and Mgt3, respectively. The phylogenetic analysis showed that Mgt1 grouped with *S. cerevisiae* mitochondrial Mg^2+^ transporters, Mgt2 had a close evolutionary relationship with the vascular Mg^2+^ transporter, and Mgt3 was similar to plasma membrane Mg^2+^ transporters (Figure 1b). Because the gene expression of many metal transporters is regulated by environmental metal levels, real‐time PCRs were carried out to measure the endogenous expression levels of *MGT1*,* MGT2,* and *MGT3* in response to elevated Mg^2+^ conditions (Figure 1c). Our data showed that the expression of *C. neoformans* Mg^2+^ transporter‐encoding genes was not regulated by environmental Mg levels.

To determine the function of *C. neoformans* Mg^2+^ transporters in maintaining Mg^2+^ balance, Mg^2+^ transporter knockout strains were generated using the targeted homologous integration method, to obtain *mgt1Δ*,* mgt3Δ,* and *mgt1Δ mgt3Δ*. In our hands, we could not disrupt *MGT2* after multiple attempts, implying an essential function for *MGT2* under normal yeast growth conditions. We therefore substituted the *MGT2* promoter with a *C. neoformans* galactose‐regulated promoter (*GAL7,* CNAG_06052: the gene coding for galactose‐1‐phosphate uridyl transferase), which was inserted before the start codon of the *MGT2* ORF. This generated a *GAL7p‐MGT2* strain in which the expression of *MGT2* was activated by galactose and repressed by glucose (Figure 1d). A DNA probe from the *GAL7* promoter was used for Southern blot analysis to evaluate the precise integration of the *GAL7* promoter cassette in the genome (Figure 1e). For the wild‐type strain, a 2400‐bp band was detected, representing the wild‐type *GAL7* promoter with the *Stu*I‐*Sac*II restriction‐digested fragment. The correct *GAL7* cassette integrations yielded a smaller band of 1800 bp. The Southern blot analysis demonstrated successful construction of the *GAL7p‐MGT2* strain in almost all transformation isolates (Figure 1e).

**Figure 2:**
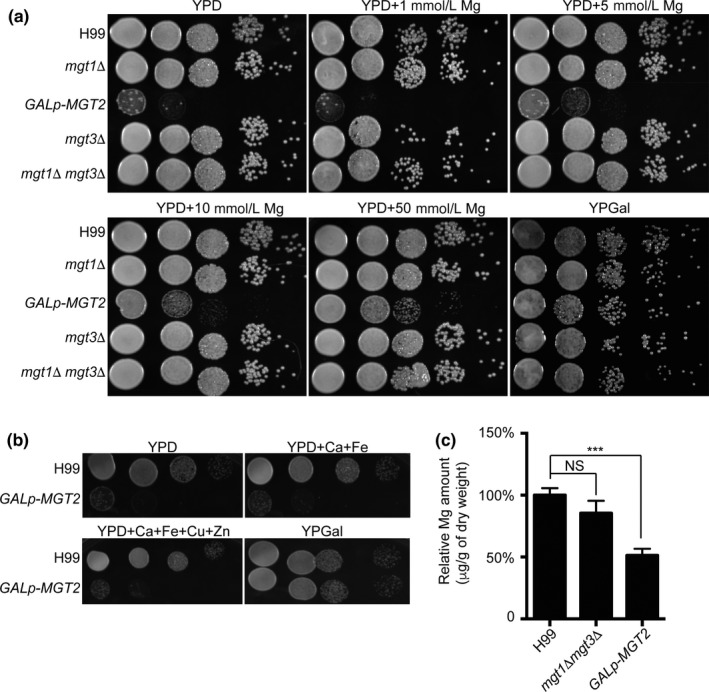
The growth phenotypes of Mg transporter mutants. (a) Cell growth assay on the Mg transporter mutants following Mg exposure. Overnight galactose liquid cell cultures of the wild‐type H99, *mgt1Δ, GAL7p‐MGT2, mgt3Δ,* and *mgt1Δ mgt3Δ* were diluted in PBS to an *A*_*600*_ of 1.0. Ten‐fold serial cell dilutions were spotted onto YPGal agar, YPD agar, or YPD agar supplemented with 1 mmol/L, 5 mmol/L, 10 mmol/L, or 50 mmol/L MgSO_4_. Plates were incubated at 30°C for 3 days before photographs were taken. (b) Cell growth assay of Mg transporter mutants following exposure to other metals. The spotting assay was performed as described in Figure 2a, except YPD agar supplemented with 50 mmol/L Ca, 200 μmol/L Fe, 1 mmol/L Cu, and 200 μmol/L Zn was used. (C) Atomic absorption spectroscopy analysis of Mg transporter mutants. Overnight galactose liquid cell cultures of the wild‐type H99, *GAL7p‐MGT2,* and *mgt1Δ mgt3Δ* strains were diluted in YPD medium to an *A*_*600*_ of 1.0, and the cultures were incubated for 6 hr. Cells were collected, washed, and placed in preweighed Eppendorf tubes. Tubes were then dried in an oven overnight. Atomic absorption spectroscopy was performed as described in the Material and Methods. Relative Mg concentrations were calculated. Four biological samples were used for the analysis. Standard deviations were determined, and the Student's *t*‐test was performed. Triple asterisks represent *p* < .001 and NS indicates no statistical significance

Using the spotting assay, the *GAL7p‐MGT2* strain showed severe growth defects when glucose was added to suppress the activity of the galactose promoter in the medium. This observation was in agreement with the difficulties related to generating an *mgt2Δ* strain (Figure 2a). The *mgt1Δ*,* mgt3Δ,* and *mgt1Δ mgt3Δ* strains had similar cell growth rates compared to the wild‐type strain. The cell growth of the *GAL7p‐MGT2* strain was rescued when Mg was supplemented into the medium, but not with other metals (Figure 2a and b). Cumulatively, our data demonstrated that Mgt2 functions as a specific Mg transporter in *C. neoformans*. We speculated that the impairment in cell growth was due to an intracellular Mg deficiency. Therefore, atomic absorption spectroscopy was used to measure cellular Mg levels. Our data showed a 50% reduction in the Mg^2+^ content in the *GAL7p‐MGT2* strain (Figure 2c). These results strongly suggested that the Mgt2 protein is a critical *C. neoformans* Mg transporter involved in intracellular Mg accumulation.

### 3.2 Mgt2 is an Mnr2 homolog

**Figure 3:**
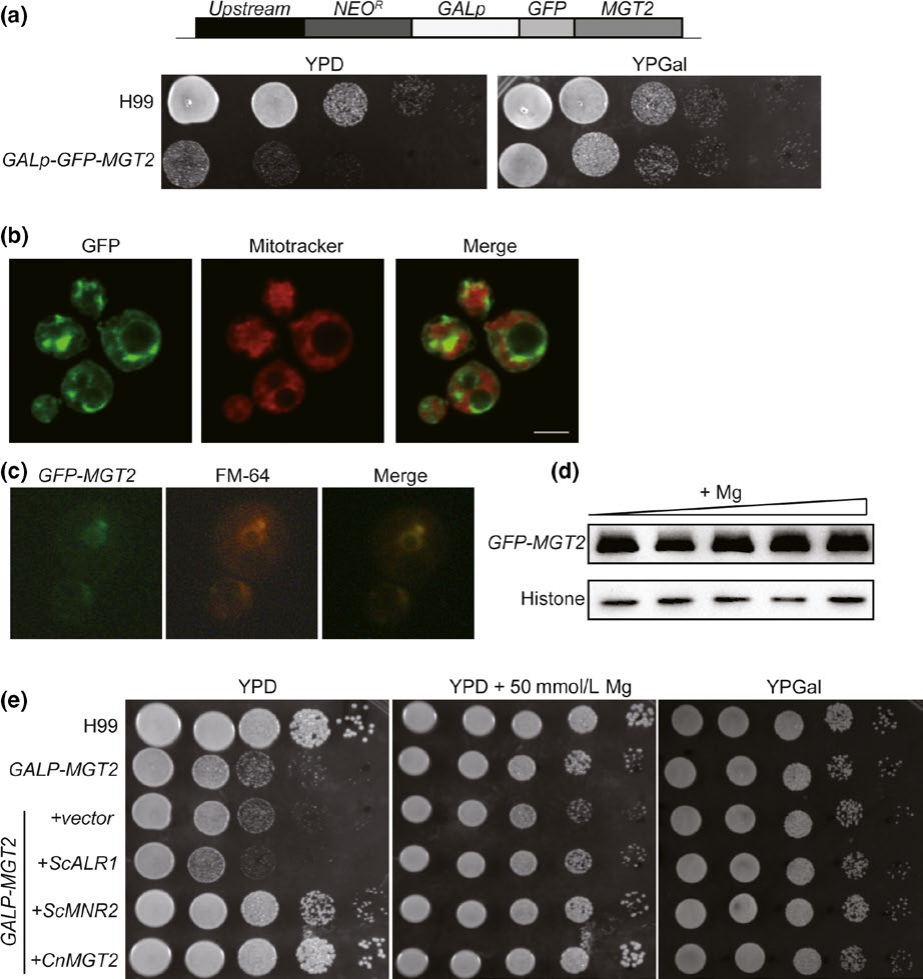
*C. neoformans* Mgt2 is a vacuolar Mg transporter. (a) Schematic for the construction of *GAL7p‐GFP‐MGT2*. A *NEO* ^*R*^ *‐GAL7p‐GFP* cassette was integrated to replace the *MGT2* endogenous promoter. The activation of *GFP‐MGT2* expression is driven by galactose. A spotting assay was performed as described in Figure 2a to confirm the strain. (b) Subcellular localization analysis of Mgt2. Cells were grown in YPGal medium supplemented with 200 μmol/L EDTA. Cells were washed twice with PBS and then stained with Mitotracker. Visualization was performed using confocal microscopy (Leica TCS‐SP8). (c) Subcellular localization analysis of Mgt2. Cells were prepared as described above, except that cells were stained with FM4‐64. (d) Expression of Mgt2‐GFP protein in high Mg. Cells were grown in YPGal medium supplemented with 0 mmol/L, 1 mmol/L, 2 mmol/L, 5 mmol/L, 10 mmol/L of MgSO_4_. Protein samples were separated and immunoblot assays were performed using anti‐GFP and antihistone H3 antibodies. (e) Complementation of the *GAL7p‐MGT2* strain phenotype by *S. cerevisiae* Mg transporter genes. *C. neoformans* expression vectors harboring *S. cerevisiae ALR1, MNR2,* or *C. neoformans MGT2* were transformed into the *GAL7p‐MGT2* strain. The resulting strains were cultured, diluted, and spotted onto YPD agar, YPD agar supplemented with 50 mmol/L MgSO_4_, or YPGal agar. The plates were incubated at 30°C for 3 days before the photographs were taken

Our phylogenetic analysis demonstrated that Mgt2 is a putative Mg^2+^ transporter and shares significant sequence similarity to Mnr2 from *S. cerevisiae* (Figure 1b). To assess whether Mgt2 functionally resembles Mnr2, a *GAL7p‐GFP‐MGT2* strain was generated. This strain expressed GFP‐Mgt2 chimeric protein under the regulation of a galactose‐inducible promoter (Figure 3a). The *GAL7p‐GFP‐MGT2* strain generated similar growth phenotypes as that of the *GAL7p‐MGT2* strain on YPD (Yeast Peptone Dextrose), and the growth phenotype was complemented in the presence of galactose (Figure 3a), demonstrating that this was a function specific to GFP‐Mgt2 protein. Confocal microscopy was used to show that GFP‐Mgt2 was localized to the vacuolar membrane (Figure 3b). We also observed GFP signal in unidentified intracellular spaces. Because *S. cerevisiae* Mg^2+^ transporters have been shown to localize to the plasma membrane, vacuolar membrane, and mitochondria (Bui et al., 1999; Cui et al., 2015; Pisat et al., 2009), we employed a mitochondrial staining method using MitoTracker to ascertain whether Mgt2 was a mitochondrial‐localized Mg^2+^ transporter in *C. neoformans*. However, no colocalization of Mgt2 and mitochondria was observed, indicating that Mgt2 is not a mitochondrial‐associated Mg^2+^ transporter (Figure 3b). A vacuolar specific dye, FM4‐64, was used to stain the *GFP‐MGT2* strain. Fluorescent signals corresponding to FM4‐64 and GFP were colocalized on the vacuolar membrane, demonstrating that the Mgt2 is a vacuolar Mg^2+^ transporter (Figure 3c). An immunoblot assay was performed to measure the protein stability of Mgt2 in response to exogenous Mg^2+^ levels. Our data demonstrated stable protein expression of Mgt2 protein under high Mg^2+^ concentrations (Figure 3d). To further address Mgt2 function, *S. cerevisiae MNR2* or *ALR1* were cloned into a *C. neoformans* expression vector containing the constitutive promoter *GPD1p*. Vectors expressing *S. cerevisiae MNR2* or *ALR1* were transformed into the *C. neoformans GAL7p‐MGT2* strain. Fungal cells were spotted onto YPD agar medium. This revealed that the *S. cerevisiae MNR2* gene functionally complemented the growth phenotype of the *GAL7p‐MGT2* strain on YPD, whereas *ScALR1* did not (Figure 3e). These data strongly suggested that *C. neoformans* Mgt2 is a vacuolar Mg^2+^ transporter that functionally analogous to *S. cerevisiae* Mnr2.

### 3.3 Mgt2 modulates gene expression independent of Mg

**Figure 4:**
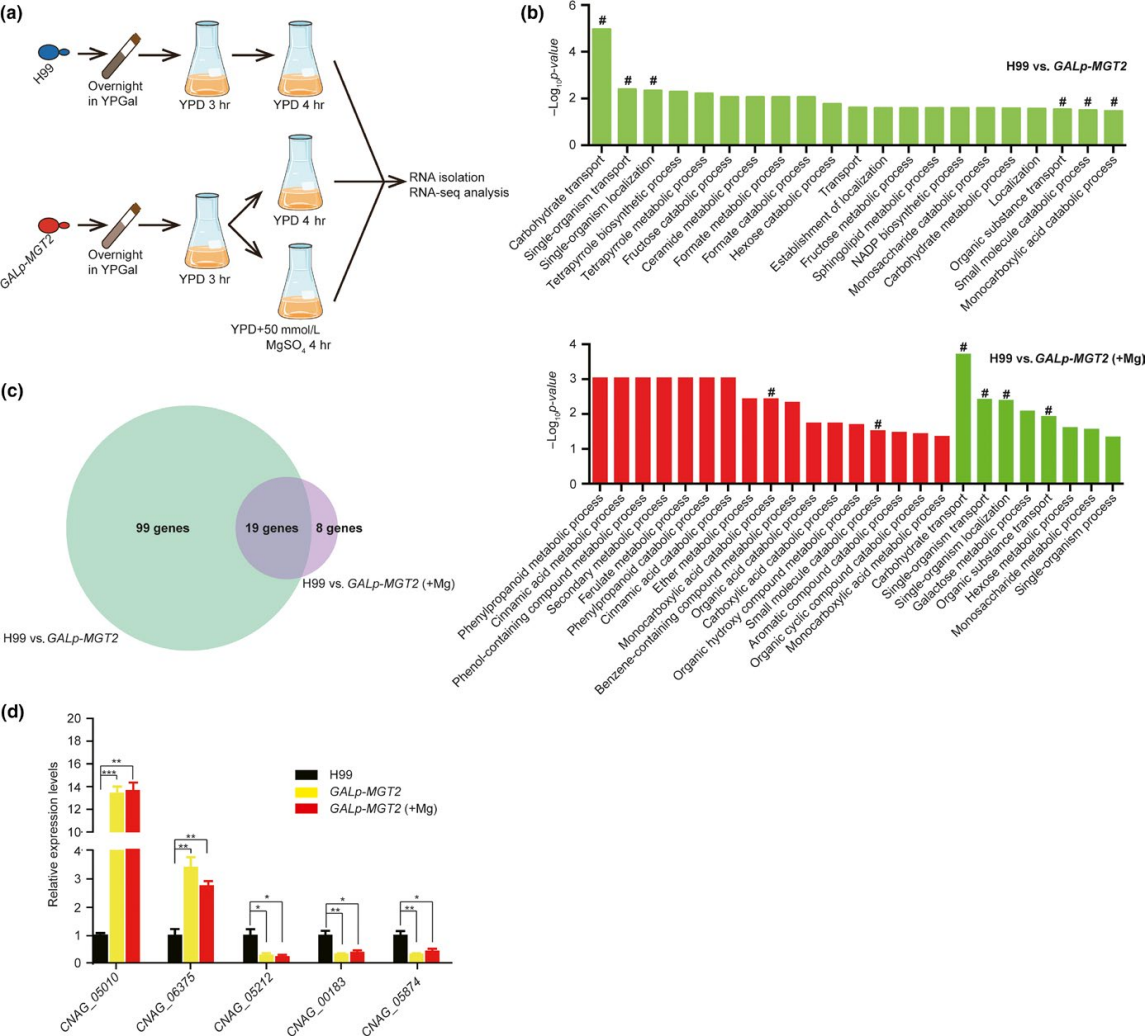
Transcriptomic analysis of the *Gal7p‐MGT2* strain. (a) Strategy for performing the transcriptomic analysis on wild‐type and *GAL7p‐MGT2* strains. Overnight YPGal cultures were diluted to an *A*_*600*_ of 1.0 in YPD medium. The YPD cultures were incubated at 30°C for 3 hr to repress the activity of the *GAL7* promoter in the *GAL7p‐MGT2* strain. For the *GAL7p‐MGT2* strain, the YPD culture was divided in two, and MgSO_4_ was added into one portion at a final concentration of 50 mmol/L. All cultures were incubated for additional 4 hr before RNA was isolated. Three biological replicates were carried out. B Gene ontology analysis. Gene ontology was performed using TopGO (Huber et al., 2015). GO terms were downloaded at http://www.bioconductor.org/packages/release/bioc/html/topGO.html. Significantly altered GO terms (classical fisher test, *p < *.05) are shown on the plot. Red bars indicate upregulation in the *GAL7p‐MGT2* strain and green bars indicate downregulation in the *GAL7p‐MGT2* strain. Hash signs show the unchanged GO terms after Mg treatment. (c) Venn diagram for transcriptomic analysis. Repressing *MGT2* causes 118 genes to be differentially expressed compared to the wild‐type strain H99. Repressing *MGT2* in the presence of exogenous Mg causes 27 genes to be differentially expressed compared to the wild‐type strain H99. Nineteen genes demonstrated similar expression dynamics in both growth conditions. (d) Real‐time PCR confirmation of transcriptomic data. Cells were grown as described in Figure 4a, and RNA was isolated, digested with DNase I, and reverse‐transcribed. Random genes were selected from 19 overlapping genes. Primers are listed in Table S1. *Actin* was used as a normalization control. Three biological replicates were performed. The Student's *t*‐test was performed to determine statistical significance (***p <* *.005, **p <* *.01, *p <* *.05).

The *mgt2* knockout strain growth phenotype was complemented when exogenous Mg was supplemented in the growth medium. However, cell growth in the presence of Mg was only partially rescued, even at a very high level of Mg^2+^ (Figure 2a). This growth phenotype suggests that Mgt2 influences normal cell growth via a Mg^2+^‐independent pathway. To explore this hypothesis, we performed a high‐throughput transcriptome analysis using RNA sequencing (RNA‐seq). We compared the transcriptomes among the three conditions: wild‐type cells in YPD medium, *GAL7p‐MGT2* cells in YPD medium, and *GAL7p‐MGT2* cells grown in YPD medium supplemented with 50 mmol/L MgSO_4_ (Figure 4a). In our data, repressing the expression of *MGT2* resulted in differential expression of 118 genes. Of these, the expression of 84 and 34 genes was down or upregulated in the *GAL7p‐MGT2* cells, respectively (Table S2). Among all differentially expressed genes, expression of *MGT2* was the most significantly downregulated in the *GAL7p‐MGT2* cells, demonstrating the accuracy of our RNA‐seq data (Table 1, Table S2 and S3). We observed that genes encoding deacetylase, mitochondrial alternative oxidase, alternative cyclin Pcl12, Fe‐S cluster assembly factor, and proteins involved in sugar metabolism and transport were significantly downregulated. Genes encoding ferric‐chelate reductase, sulfite transporter, septin ring protein, and glucose‐methanol‐choline oxidoreductase were significantly upregulated (Table S2). On the other hand, supplementing Mg into the medium of *GAL7p‐MGT2* cells only caused 27 genes to be differentially expressed. Of these, expression of 7 genes and 20 genes was up or downregulated, respectively (Table S3). We detected that genes encoding alcohol dehydrogenase, alternative cyclin pcl12, carnitine O‐acetyltransferase, and alternative oxidase were downregulated in the *GAL7‐MGT2* strain when grown in high or low Mg conditions. Gene ontology analysis demonstrated that the addition of Mg into the medium of the *GAL7p‐*MGT2 strain caused dramatic changes in the GO enrichment (Figure 4b) compared to that of *GAL7p‐MGT2* cells in YPD only. However, four GO terms remained unchanged in both conditions, regardless of Mg treatment, and these GO terms were significantly enriched in carbohydrate transport, single‐organism transport, single‐organism localization, and organic substance transport (Figure 4b). Two processes were differentially regulated upon the Mg^2+^ treatment, and these GO terms were enriched in monocarboxylic acid catabolic process and small molecule catabolic process. The expression of 19 genes remained constant in *GAL7p‐MGT2* cells in YPD in the presence or absence of exogenous Mg (Figure 4b and Table 1), and this included *MGT2* itself. Real‐time PCR was performed for five randomly selected putative target genes of the 19‐gene cohort to evaluate the RNA‐seq data, and expression regulation patterns resembled those obtained in the RNA‐seq analysis (Figure 4c).

**Table 1:** RNA‐seq analysis of differentially expressed genes in the *GAL7‐MGT2* strain supplemented with or without MgSO_4_

Gene ID	Log_2_(*MGT2* ‐v‐ H99)^a^	Log_2_(*MGT2 *+* *Mg ‐v‐ H99)^b^	Description
CNAG_03502	−4.28	−3.44	Magnesium transporter
CNAG_02489	−1.20	−2.37	Alcohol dehydrogenase
CNAG_03772	−0.92	−2.28	Glucose transporter
CNAG_07788	−1.62	−1.98	Unknown
CNAG_05212	−2.08	−1.72	Unknown
CNAG_07819	−1.33	−1.64	Unknown
CNAG_05487	−1.65	−1.61	Unknown
CNAG_07845	−1.84	−1.41	Unknown
CNAG_05874	−1.55	−1.20	Unknown
CNAG_00679	−1.32	−1.18	Unknown
CNAG_00183	−1.65	−1.09	Alternative cyclin Pcl12
CNAG_01370	−0.92	−1.01	Unknown
CNAG_06551	−0.84	−0.89	Carnitine O‐acetyltransferase
CNAG_01065	−0.78	−0.72	Unknown
CNAG_00162	−0.82	−0.64	Alternative oxidase
CNAG_00664	0.91	1.16	Unknown
CNAG_02392	0.98	1.33	Unknown
CNAG_06375	2.14	2.25	Unknown
CNAG_05010	2.80	3.23	Unknown

a RNA‐seq analysis was performed and differential gene expression was compared between the wild‐type and *GAL7‐MGT2* strains. Differential gene expression is represented by binary log of the fold change. Three biological replicates were performed. The full gene list is available in Table S2.

b RNA‐seq analysis was performed and differential gene expression was compared between the wild‐type and *GAL7‐MGT2* strains (supplemented with 50 mM MgSO_4_). Differential gene expression is represented by binary log of the fold change. Three biological replicates were performed. The full gene list is available in Table S3.

### 3.4 Mgt2 influences *C. neoformans* survival in the lungs

**Figure 5:**
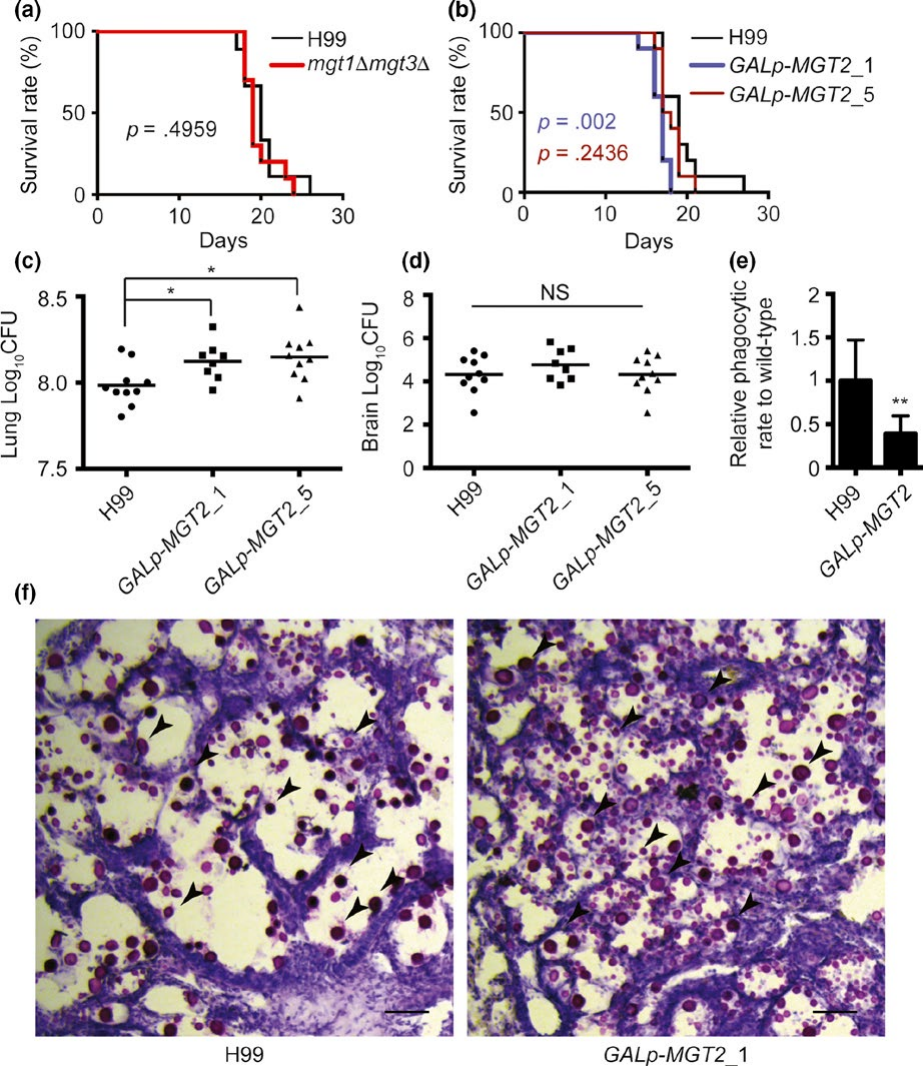
Analysis of *C. neoformans* Mg transporter mutants in mice. (a) Survival analysis of the wild‐type and *mgt1Δ mgt3Δ* strains. Twenty 4–6‐week‐old female C57BL/J mice were used for the analysis. Overnight fungal cell cultures were washed twice with PBS and diluted with PBS. A PBS suspension containing 10^5^ cells of the wild‐type or *mgt1Δ mgt3Δ* strain were used for the infection of mice. Animal survival rates were recorded. (b) Survival analysis of the wild‐type and *GAL7p‐MGT2* strains. Twenty 4–6‐week‐old female C57BL/J mice were used for the analysis. Overnight YPGal fungal cell cultures were washed twice with PBS and diluted in YPD medium. The YPD cultures were incubated for 6 hr to repress the *GAL* promoter. Cells were then washed and diluted. A PBS suspension containing 10^5^ cells of the wild‐type or *mgt1Δ mgt3Δ* strain were used for infection in mice. Animal survival rates were recorded. (c) Fungal burden analysis in the lungs. Two independent *GAL7p‐MGT2* strains were used to infect mice as described in Figure 5b. Fourteen days post infection, mice were killed, and CFUs from the lung tissues were analyzed. (d) Fungal burden analysis in the brain. The brain fungal burden tests were performed as described in Figure 5c. ANOVA was used for statistical analysis (*p <* *.05). (e) Phagocytic assay of *mgt2* mutant. YPGal fungal cell cultures were washed twice with PBS, and then mixed with RAW264.7 cells in DMEM medium supplemented with 10% FCS. Extracellular fungal cells were washed with PBS buffer. Internalized and membrane‐bound fungal cells were counted. Relative phagocytic rates were calculated. The graph represents three biological replicates, and over 150 macrophages were counted for each replicate. The Student's *t*‐test was performed to determine statistical significance (**p <* *.01). (f) Histopathological analysis of *C. neoformans*‐infected lung tissues. Lung tissues from H99‐ and *Gal7p‐MGT2*‐infected mice were isolated, fixed, and stained with mucicarmine or H&E. Arrows indicates strained *C. neoformans* cells

Because Mg^2+^ is an essential metal and the *mgt2* mutant showed striking growth defects, we hypothesized that Mgt2 participates in regulating fungal virulence in mice. C57BL/J mice were infected with *mgt1Δ mgt3Δ* or *GAL7p‐MGT2* strains (precultured in YPD medium), and the survival rates were recorded (Figure 5a). Mice infected with *mgt1Δ mgt3Δ* had similar death rates as the wild‐type. Surprisingly, mice infected with two independent *GAL7p‐MGT2* strains had earlier death rates, but these data displayed poor reproducibility (Figure 5b). In the colony‐forming unit (CFU) analysis, we detected increased levels of fungal proliferation in two independent *GAL7p‐MGT2* strains in the lungs (Figure 5c). This was subsequently recapitulated by the lung tissue mucicarmine stain in which more *GAL7p‐MGT2* cells were observed in lung tissues compared to the lung tissue infected with the wild‐type strain (Figure 5f), with no significant changes observed for lung mass (data not shown). Furthermore, using the murine macrophage cell line RAW264.7, we showed that the *mgt2* mutant inhibited the rate of phagocytisis, compared to the wild‐type cells (Figure 5e), suggesting that Mgt2 may participate in capsule formation (discussed below). A comparison of the CFUs from brain tissues between H99 and *GAL7p‐MGT2* cells showed no significant difference (Figure 5d).

**Figure 6:**
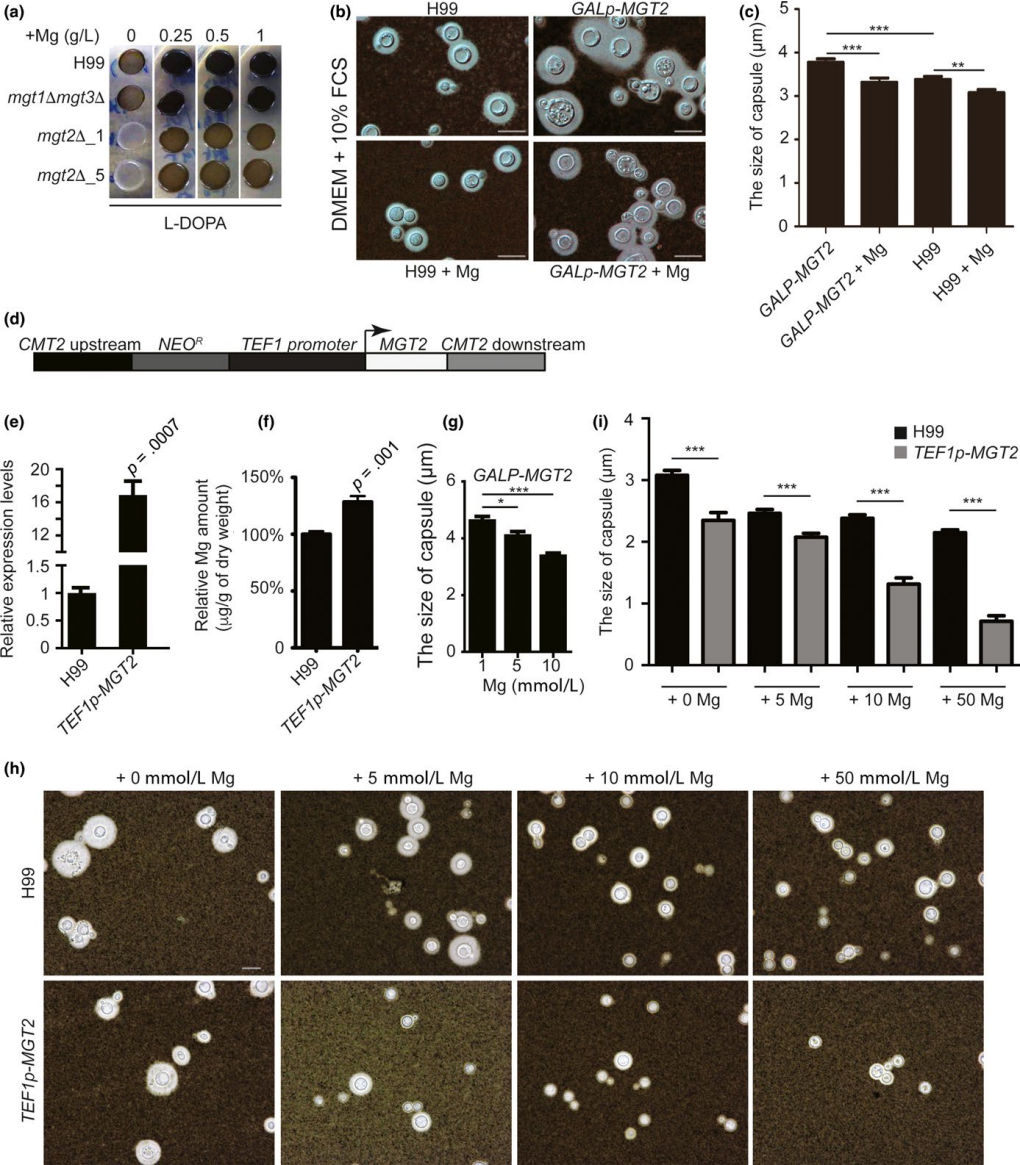
*C. neoformans* Mgt2 modulates melanin production and capsule structure. (a) Melanin formation in *C. neoformans* Mg transporter mutants. YPGal overnight cell cultures of H99, *mgt1Δ mgt3Δ*, and two independent *Gal7p‐MGT2* strains were diluted and subcultured in YPD. Cells were then washed, diluted, and spotted onto L‐DOPA plates supplemented with various concentration of MgSO_4_. The plates were incubated at 37°C until black pigment developed. Photographs were taken at day 3 of incubation. (b) Capsule formation in the *GAL7p‐MGT2* strain. YPGal overnight cell cultures of H99 and *GAL7p‐*MGT2 strains were diluted in DMEM supplemented with 10% FCS, with or without exogenous Mg. Cultures were incubated at 37°C (5% CO_2_) for 2 days. Pictures were taken using a Nikon Eclipse Ni‐E microscope. **(c)** Capsule structure quantifications in the *GAL7p‐MGT2* strain in DMEM medium. The thickness of capsule structure was measured and quantified. At least 50 fungal cells from Figure 6b were measured. The Student's *t*‐test was performed (***p <* *.005, **p < .01). (d) Model for the overexpression of Mgt2. A linearized plasmid containing a *NEO*‐resistant gene, a constitutive promoter from *C. neoformans* (*TEF1*), *CMT2* flanking sequences, and the *MGT2* ORF were introduced into the H99 strain. The integration of the overexpression cassette occurred at the *CMT2* locus. *MGT2* expression was driven by the *TEF1* promoter. (e) Real‐time PCR quantification of *MGT2* in the *TEF1p‐MGT2* strain. RNA samples were isolated from the H99 and *GAL7p‐MGT2* strains and reversed‐transcribed into cDNA. Real‐time PCR on *MGT2* cDNA was performed. Four biological replicates were performed. (f) Atomic absorption spectroscopy analysis in the *TEF1p‐MGT2* strain. The assay was performed as described in Figure 2c. (g) Capsule structure quantifications in the *GAL7p‐MGT2* strain in ACSF medium. At least 50 fungal cells were measured. The Student's *t*‐test was performed (***p <* *.005, *p < .05). (h) Capsule structure quantification in the *TEF1p‐MGT2* strain. Overnight YPD cell cultures were diluted in ACSF medium and supplemented with various concentrations of MgSO_4_. Cultures were incubated at 37°C for 3 days. Pictures were taken using a Nikon Eclipse Ni‐E microscope. (i) Capsule structure quantifications in the *TEF1‐MGT2* strain. The thickness of the capsule structure from Figure 6f was measured and quantified. At least 50 fungal cells from Figure 6f were measured. The Student's *t*‐test was performed (***p <* *.005)

To gain further insights into the mechanism by which Mgt2 influences fungal proliferation in the lung, we analyzed melanin and capsule formation in the *GAL7p‐MGT2* strains. As shown in Figure 6a, melanin formation in the wild‐type cells required Mg^2+^. The *GAL7p‐MGT2* strains demonstrated severe melanin production defects. The addition of Mg^2+^ only partially rescued the pigmentation defects. We also tested the *mgt1Δ mgt3Δ* strain, and it showed similar melanin production deficits when compared with the wild‐type strain (Figure 6a). Our data demonstrated that Mg^2+^ is a critical element for melanin formation in *C. neoformans*, and Mgt2 is required.

Next, we examined capsule formation in Mg^2+^ transporter mutants in DMEM (Dulbecco's Modified Eagle Medium, high glucose) supplemented with 10% fetal calf serum (FCS) (Figure 6b). We observed a modest increase in capsule thickness in the *GAL7p‐MGT2* strain compared to the wild‐type strain (Figure 6c). The addition of Mg^2+^ reduced the size of the capsule structure. To extend these observations, we constructed an Mgt2 overexpression strain in which *MGT2* expression is under the control of the constitutive promoter, *TEF1* (Figure 6d). To avoid random integration in the genome, the construct was designed to target the metallothionein 2 locus (*CMT2*) because previous studies demonstrated that the disruption of *CMT2* had no effect on the expression of virulence factors (Ding et al., 2013). The expression of *MGT2* was measured using real‐time PCR, which demonstrated a 15‐fold increase in gene expression compared to that of the wild‐type strain (Figure 6e). A significant increased intracellular Mg^2+^ concentration was detected in the *TEF1‐MGT2* strain (Figure 6f). Since previous studies showed that different capsule induction environments result in the variations in capsule thickness (Rathore et al., 2016), we employed ACSF (artificial cerebrospinal fluid) medium to evaluate the capsule structure in the *GAL7p‐MGT2* and *TEF1p‐MGT2* strains. Significant reductions in the size of the capsule structure were observed in the *GAL7p‐MGT2* strain in ACSF medium supplemented with a high concentration of Mg^2+^ (Figure 6g). These effects were similar to those of DMEM and ACSF media on capsule formation in the *GAL7p‐MGT2* mutant. In contrast to the *GAL7p‐MGT2* strain, the *TEF1p‐MGT2* strain displayed a reduction in capsule thickness, and a greater reduction was observed in the presence of exogenous Mg^2+^ (Figure 6h and i). These results clearly demonstrated that expression of *MGT2* has a negative effect on capsule production through the regulation of intracellular Mg^2+^ contents, increasing the cell survival of the *GAL7‐MGT2* mutant strain in the lung tissue.

## 4 DISCUSSION

*C. neoformans* is a deadly human fungal pathogen, yet clinical antifungal therapies are ineffective. Many studies have demonstrated the important function of metal homeostasis in modulating *C. neoformans* virulence and host defenses (Ding et al., 2014; Samanovic, Ding, Thiele, & Darwin, 2012). Fe has been widely accepted as a key regulator of pathogenicity in major human fungal pathogens (Bairwa et al., 2017; Ding et al., 2014; Jung et al., 2006; Lian et al., 2005; Nyhus et al., 1997; Saikia, Oliveira, Hu, & Kronstad, 2014). In *C. neoformans*, mutations in genes associated with Fe acquisition often lead to dramatic attenuations in fungal virulence. A *C. neoformans* GATA‐type transcription factor, Cir1, is required for the regulation of expression of genes that are essential for Fe and Cu homeostasis. Moreover, important virulence factor genes, such as genes involved in the capsule and melanin biosynthesis processes, are also controlled by Cir1 (Jung et al., 2006). Using metabolomic analysis, it was demonstrated that Cir1 modulates the glycolytic pathway, ergosterol processes, and inositol metabolism. Other metal regulators, including HapX and Leu1, also contribute to metal homeostasis and regulate virulence in *C. neoformans* (Choi, Kim, Kim, Jung, & Lee, 2012; Do et al., 2015, 2016; Jung et al., 2010).

Numerous studies suggest that metal acquisition machinery has an essential function in *C. neoformans* virulence. Indeed, disrupting the high‐affinity Fe^2+^ permease Cft1 in *C. neoformans* significantly attenuates fungal virulence and fungal burden in animal models as a result of the poor adaptation of mutant cells during Fe limitation in the host. However, the disruption of Cft2 has no effect on fungal proliferation in the host (Han, Do, & Jung, 2012; Kim et al., 2012). Cu has been suggested to be an essential metal for *C. neoformans* because of its function in melanin formation, capsule production, respiration, and as an antioxidant agent cofactor (Chun & Madhani, 2010; Ding et al., 2013; Jiang et al., 2009; Mauch, Cunha, & Dias, 2013; Walton et al., 2005; Waterman et al., 2007, 2012; Zhang et al., 2016). Recent studies of Cu homeostasis focused on two Cu^+^ transporters, *CTR1* and *CTR4*. It has been shown using two versions of a gene knockout system that a *ctr4* mutant demonstrates high fungal burden in the lungs (Sun et al., 2014; Zhang et al., 2016). Meanwhile*,* the *ctr1* and *ctr4* double mutant has melanin defects, and this strain shows an increased fungal burden and brain dissemination (Sun et al., 2014).

Mg^2+^ is one of the most abundant divalent metal elements required for life, but its potential function in regulating fungal pathogenicity remains uninvestigated. Previous in vitro studies demonstrated that Mg^2+^ is an important element in the regulation of biofilm formation and capsule production in *C. neoformans* (Robertson, Wolf, & Casadevall, 2012). The addition of ethylenediaminetetraacetic acid (EDTA) reduces biofilm growth and vesicular secretion from the cell. However, these inhibitory effects are the result of divalent cation deficiency, rather than a Mg^2+^‐specific factor. This is because the supplementation of either Mg^2+^ or Ca^2+^ could rescue the inhibitory effects of EDTA. Interestingly, recent studies showed that Mg^2+^ has a potential influence on capsule structure in clinical *C. neoformans* isolates, but the molecular mechanism remains unknown (Rathore et al., 2016).

In our study, three Mg^2+^ transporter gene orthologs are identified in the genome of *C. neoformans*, based on BLAST searches using *S. cerevisiae* Mg transporters. Many key players involved in metal homeostasis are regulated transcriptionally by metal concentrations in the growth medium. For example, gene expression of *CTR1* and *CTR4* from *C. neoformans* decreases upon treatment with Cu^+^ (Ding et al., 2011). Unlike the response to other metals, the gene expression and protein levels of Mg transporters in *C. neoformans* are highly stable after supplementation with Mg^2+^ ions, demonstrating significantly different regulation patterns at the transcriptional and posttranslational level for Mg homeostasis. *S. cerevisiae* Alr1 and Alr2 are two Mg importers localized to the plasma membrane (Graschopf et al., 2001). Knocking out both genes impairs *S. cerevisiae* cell growth even at relatively high Mg concentrations (4 mmol/L Mg^2+^ supplementation in the growth medium), indicating the essential function of these Mg^2+^ importers in fungal biology (Graschopf et al., 2001; Pisat et al., 2009). Interestingly, the deletion of *C. neoformans MGT3* (an ortholog of plasma membrane Mg^2+^ transporters in *S. cerevisiae*) causes no Mg‐dependent growth phenotypic difference compared to the wild‐type strain. Normal growth rates are observed in the *mgt1* mutant, which is an ortholog of *S. cerevisiae* mitochondrial Mg transporters. A double mutant for *MGT1* and *MGT3* also has a comparable phenotype as the wild‐type *C. neoformans* strain, and no defects in cell growth, melanin, capsule formation, or virulence are observed in the animal model. These results imply that the *C. neoformans* genome encodes other Mg^2+^ transport factors that facilitate Mg^2+^ import into the cytosol and mitochondria.

A *C. neoformans MGT2*‐repressing mutant has a reduced intracellular Mg^2+^ content and cell growth defects under normal growth conditions, resembling the phenotype of *S. cerevisiae* (Pisat et al., 2009). Moreover, *S. cerevisiae* Mnr2 functionally complements *C. neoformans* Mgt2, and the *C. neoformans* Mgt2 has vacuolar protein localization. Taken together, our work demonstrates the conserved function of Mnr2 in *C. neoformans* and *S. cerevisiae*. Because of the striking cell growth defects in the *mgt2* mutant, we speculate that the *mgt2* mutant strain would have an impaired virulence and fungal burden in lung tissue. Surprisingly, our animal infection studies showed minor increases in mortality in the animals infected with the *mgt2* mutants, in addition to significant inductions in the fungal burden in the lung tissue. Further analysis indicated that the increased fungal proliferation in the host results from an enlarged capsule structure when expression of Mgt2 was repressed, leading to the inhibition of macrophage phagocytosis. In agreement with the observation of capsule enlargement in the *mgt2* mutant, the overexpression of Mgt2 resulted in less capsule production. Furthermore, the reduction in the capsule is inversely correlated with the addition of exogenous Mg^2+^ ions, demonstrating a direct connection between Mg^2+^ homeostasis and capsule production in *C. neoformans*. This highlights the important function of Mgt2 in controlling the capsule structure. Despite our data showing melanin formation defects in the Mgt2‐repressing strain, pigment development is not fully complemented when exogenous Mg is supplemented, indicating that both Mg^2+^ and Mgt2 are required for proper melanin production in *C. neoformans*.

It is interesting that Mgt2 has a positive effect on the cell growth, which is independent of intracellular Mg level. This result indicates the potential novel functions of Mg^2+^ transporters in *C. neoformans*. In fact, Mg^2+^ transporters have been demonstrated to be involved in the RNA group II splicing process in *S. cerevisiae* (Gregan et al., 2001; Sponder et al., 2010). While it is still unclear what the regulation mechanism is in, it is possible that the Mgt2‐repressing strain dramatically dampens the intracellular Mg levels, and thus impairs the RNA splicing process in *C. neoformans*, thus blocks the gene expression. Using transcriptomic analysis, we show here that *C. neoformans* Mgt2 differentially regulates the expression of 19 genes, regardless of Mg concentration. We observed genes encoding alternative cyclin Pcl12 and carnitine O‐acetyltransferase were decreased in expression when *MGT2* was repressed. Previous studies have demonstrated the critical functions of Pcl12 and carnitine O‐acetyltransferase in the regulation of cell cycle progression and cell morphology (Brunner, Kramar, Denhardt, & Hofbauer, 1997; Flor‐Parra, Castillo‐Lluva, & Perez‐Martin, 2007; Perez‐Martin & Castillo‐Lluva, 2008). Therefore, it is most likely that the growth defects of the *GAL7‐MGT2* strain after Mg treatment is due to the inhibition of gene expression of cell proliferation regulators.

Despite a previous study using semiquantitative RT‐PCR showing that Mg^2+^ influences the expression of genes involved in capsule production, we do not detect capsule‐related RNA expression differences in our RNA‐seq analysis. This is probably because different strain isolates were used in each study, causing different responses to metal ions (Sun et al., 2014; Waterman et al., 2012; Zhang et al., 2016). In fact, *C. neoformans* is a fast‐evolving human fungal pathogen. Comparative genomic analyses have demonstrated inconsistent phenotypic variations in stress responses, mating and virulence factor expression among H99 lineages because of these microevolutionary processes (Bahn, Hicks, Giles, Cox, & Heitman, 2004; Geddes et al., 2016; Gong, Grodsky, Zhang, & Wang, 2014; Janbon et al., 2014; Maeng et al., 2010; Pukkila‐Worley & Alspaugh, 2004; Shen, Wang, Whittington, Li, & Wang, 2008). Most importantly, the enlargement of capsule structure and increased fungal lung proliferation in *MGT2*‐repressed cells may also be determined by other factors, because *C. neoformans* Mgt2 differentially regulates the expression of 19 different genes. Previous studies demonstrated that *C. neoformans* capsule formation is induced by low glucose conditions, via the activation of the cAMP‐PKA pathway, which is also an extremely important regulator of *C. neoformans* virulence. Interestingly, the expression of the glucose transporter (CNAG_03772) is repressed when Mgt2 expression is inhibited. It is likely that the *mgt2* mutant activates the cAMP‐PKA pathway via intracellular glucose deficiency, which in turns, causes capsule structure induction and increases lung fungal burden. Therefore, we suggest that multiple inputs are needed for the capsule development and the induction of fungal virulence in the *mgt2* mutant. Moreover, we also detected that Mgt2 regulates 13 hypothetical genes that may play important roles in *C. neoformans* capsule formation.

In conclusion, we have demonstrated an evolutionarily conserved role for a vacuolar Mg transporting protein in *C. neoformans*, and highlighted the important function of Mg^2+^ in modulating *C. neoformans* virulence factors.

## CONFLICT OF INTEREST

None declared.
